# Monocarboxylate Transporter 6-Mediated Interactions with Prostaglandin F_2α_: In Vitro and In Vivo Evidence Utilizing a Knockout Mouse Model

**DOI:** 10.3390/pharmaceutics12030201

**Published:** 2020-02-26

**Authors:** Robert S. Jones, Mark D. Parker, Marilyn E. Morris

**Affiliations:** 1 Department of Pharmaceutical Sciences, School of Pharmacy and Pharmaceutical Sciences, University at Buffalo, State University of New York, Buffalo, NY 14214, USA; jonesr45@gene.com; 2 Current Address Is Drug Metabolism and Pharmacokinetics, Genentech, Inc., South San Francisco, CA 94080, USA; 3 Department of Physiology and Biophysics, Jacobs School of Medicine and Biomedical Sciences, University at Buffalo, State University of New York, Buffalo, NY 14203, USA; parker28@buffalo.edu

**Keywords:** transporters, CRISPR/Cas9, prostaglandins, diet, monocarboxylate transporter 6, slc16a5

## Abstract

Monocarboxylate transporter 6 (MCT6; *SLC16A5*) is a recently studied drug transporter that currently has no annotated endogenous function. Currently, only a handful of compounds have been characterized as substrates for MCT6 (e.g., bumetanide, nateglinide, probenecid, and prostaglandin F_2α_ (PGF2α)). The objective of our research was to characterize the MCT6-specific transporter kinetic parameters and MCT6-specific in vitro and in vivo interactions of PGF2α. Murine and human MCT6-mediated transport of PGF2α was assessed in MCT6-transfected oocytes. Additionally, endogenous PGF2α and a primary PGF2α metabolite (PGFM) were measured in plasma and urine in Mct6 knockout (Mct6^−/−^) and wild-type (Mct6^+/+^) mice. Results demonstrated that the affinity was approximately 40.1 and 246 µM respectively, for mouse and human, at pH 7.4. In vivo, plasma PGF2α concentrations in Mct6^−/−^ mice were significantly decreased, compared to Mct6^+/+^ mice (3.3-fold). Mct6^-/-^ mice demonstrated a significant increase in urinary PGF2α concentrations (1.7-fold). A similar trend was observed with plasma PGFM concentrations. However, overnight fasting resulted in significantly increased plasma PGF2α concentrations, suggesting a diet-dependent role of Mct6 regulation on the homeostasis of systemic PGF2α. Overall, these results are the first to suggest the potential regulatory role of MCT6 in PGF2α homeostasis, and potentially other PGs, in distribution and metabolism.

## 1. Introduction

Monocarboxylate transporters (MCTs) are solute carrier proteins of the *SLC16A* family, which is comprised of fourteen isoforms [1]. While the well-characterized MCTs 1–4 have been predominantly studied for their roles as lactate-proton symporters and drug targets due to their upregulation in a wide variety of cancers [2,3,4], other isoforms have become more recently characterized within the past decade. One of which, monocarboxylate transporter 6 (MCT6; *SLC16A5*), was found to be implicated in the transport of a wide variety of drugs (i.e., bumetanide, nateglinide, probenecid, and prostaglandin F_2α_ (PGF2α)), as well as dependent on pH and membrane potential [5,6]. In addition, our lab recently characterized MCT6′s interactions with a series of commonly ingested aglycone flavonoids, suggesting its role in their potential transport and/or drug–diet interactions [7]. Gene expression data suggest that MCT6 activity plays a primary role in tissues involved primarily in drug absorption and disposition, such as the kidney, liver, and intestine [6,8,9]. However, due to the limited resources for measuring MCT6 protein expression, no reliable data is currently available investigating MCT6 membrane localization. In addition, to date, no well-characterized endogenous substrates have been annotated for MCT6, making it an orphan transporter.

Transcriptomic analyses performed in mouse liver homogenate also investigated the effects of certain dieting states on *Slc16a5* (*Mct6*) gene expression and found that it was significantly upregulated. In one study, *Slc16a5* was characterized as one of the top 40 most upregulated genes in murine liver tissue following a fenofibrate-supplemented diet for two weeks according to mean fold change [10]. In the second study, the murine hepatic transcriptome was compared between groups that were fasted for 24 h or fed a normal chow ad libitum [11]. From this study, Zhang et al. found that *Slc16a5* was upregulated ~5-fold following a fasted diet compared to a normal diet, making it one of the top 15 fold-change genes upregulated by fasting out of the 2305 genes found to be significantly regulated by food availability. More recently, Xu et al. showed that MCT6 may play a role in dietary metabolic pathways using a rat model of diabetes [12]. While the amino acid sequence identity between murine Mct6 (mMct6) and human MCT6 (hMCT6) is relatively similar (~68% according to Clustal Omega), very little data exists regarding the correlation between mMct6 and hMCT6 activity.

Prostaglandins (PGs) are important ‘hormone-like’ signaling molecules and members of the eicosanoid family. Derived from arachidonic acid metabolism, PGs are key mediators in a wide range of essential functions such as inflammation [13], blood pressure [14], and smooth muscle contraction [15]. All PGs share specific structural traits, including a 20-carbon backbone as well as a five-membered ring. Synthesized primarily via the cyclooxygenase (COX) pathway, which include the basal COX-1 and stimulatory COX-2 enzymes, PGs exhibit their tissue-dependent effects primarily via a series of G-protein coupled receptors. In addition, due to their polar, anionic nature at physiological pH, transporter-mediated distribution of PGs has been shown to play a major role in their tissue-specific absorption and efflux [16]. The major PG transporter (PGT), otherwise known as OATP2A1 (*SLCO2A1*), transports various PGs at nanomolar affinity [17,18], and is primarily expressed in the lung [19], kidney, spleen, and heart [20] as well as ocular tissue [21]. Other transporters involved in PG absorption and efflux, as well as other arachidonic acid metabolites, include the SLC22A family isoforms: organic anion transporters (OAT) OAT 1, 2 [22], 3 [23], OAT-PG [24], and members of the multidrug resistant protein (MRP) family including MRP1, MRP2 [16], and MRP4 [25]. Following transport into the cell, tissue-dependent PG clearance mechanisms often take place primarily via rapid β-oxidation in the cytosolic compartment into a number of active and inactive primary metabolites. Present in virtually all nucleated cells, PGs and their receptors have been in the spotlight due to their utility as targets in many instances where these regulatory processes are perturbed, such as inflammation [13,26], cancer [27,28], and obesity [29,30]. In particular, prostaglandin F_2α_ (PGF2α), also termed dinoprost, is a drug used to induce labor via regulation of uterine contractions. In addition, recent evidence suggests that PGF2α is involved in adipogenesis, lipogenesis, and diet-induced obesity [31,32]. Of particular interest with regards to MCT6, PGF2α was previously characterized as a substrate for human MCT6 using transfected *Xenopus laevis* oocytes [5]. Our goal in this study was to assess the contribution of MCT6 in the transport of PGF2α. We evaluated the transporter kinetic parameters (K_t_, J_max_) using both murine Mct6- and human MCT6-transfected *X. laevis* oocytes and investigated whether there were any other significant interactions with other PGs. Additionally, using a Mct6^-/-^ mouse model, we examined whether there were any changes in endogenous levels of PGF2α and its primary metabolite (13,14-dihydro-15-keto PGF2α; PGFM). The effects of diet on plasma and urinary concentrations of PGF2α and PGFM were assessed and compared with concentrations in fasted and fed animals, in order to evaluate the effects of fasting on Mct6 expression and influence on its endogenous substrate PGF2α.

## 2. Materials and Methods

### 2.1. Materials 

Prostaglandin F_2α_ and other compounds were purchased from Cayman Chemical (Ann Arbor, MI, USA). All ELISAs were also purchased from Cayman Chemical. [^3^H]PGF2α was purchased from Perkin Elmer (Waltham, MA, USA). GIBCO Leibovitz’s L-15 medium with glutamine (Cat. # 41300-039), and all Western Blotting materials were supplied by Thermo Fisher Scientific (Rockford, IL, USA). For cloning purposes, complementary DNA (cDNA) encoding for murine *Slc16a5* (NM_001080934.1), TOPO^®^ TA cloning kit, and mMESSAGE mMACHINE T7 transcription kit were purchased from Thermo Fisher Scientific. For DNA isolation, a REDExtract-N-Amp™ Tissue Polymerase Chain Reaction Kit was purchased from Sigma Aldrich (St. Louis, MO, USA). Bumetanide and probenecid were also purchased from Sigma Aldrich. The FlashGel™ System was purchased from Lonza (Portsmouth, NH, USA). All enzymes were purchased from New England Biotechnology (Ipswich, MA, USA). The Gel Extraction and PCR Purification kits were purchased from Qiagen (Valencia, CA, USA). DNA purity and concentration were verified using a NanoDrop 1000 instrument (Thermo Fisher Scientific, Rockford, IL, USA). The rabbit polyclonal anti-FLAG antibody (Cat. # ab1162) was purchased from Abcam (Cambridge, MA, USA).

### 2.2. Animals

Male Mct6^−/−^ and C57BL/6NCr (Mct6^+/+^) mice were used for the in vivo studies (Charles River, Wilmington, MA, USA). All mice were housed in cages with a 12 h light/12 h dark cycle. Animals were given free access to normal chow (Envigo 2018 Teklad global 18% protein extruded rodent diet) ad libitum and water. For the fasted groups of mice, mice were placed in individual cages without access to food for 12–15 h overnight, from which urine was collected in metabolic cages and blood was sampled the following morning via submandibular puncture. All experiments were conducted under the approval of the Institution of Animal Use and Care Committee, State University of New York at Buffalo (PROTO201800153, Approved: 18/01/2017). Mice were sacrificed via cardiac puncture and cervical dislocation. Mct6^-/-^ mice were bred and developed as described previously [33]. Wild-type (Mct6^+/+^) mice were allowed to equilibrate to a housing environment for at least one week prior to experimentation. Male mice were used for the purposes of all studies outlined.

### 2.3. Generation of the pGH19-mMct6/hMCT6 Vectors

cDNA encoding for murine *Slc16a5* (NM_001080934.1) was purchased from Thermo Fisher Scientific (GeneArt, Rockford, IL, USA). The sequence was designed and optimized to contain 5′ (*XmaI*) and 3′ (*XbaI*) restriction sites to flank the open reading frame, a Kozak consensus sequence prior to the start codon, and a C-terminal FLAG tag for immunodetection. Briefly, cDNA was amplified using 5′ and 3′ primers prior to the restriction sites using reverse transcription polymerase chain reaction (RT-PCR) on a BioRad CFX Connect™ RT System. The PCR reaction was purified, and the cDNA and previously used oocyte expression vector (pGH19) [7,34] were double-digested with *XmaI* and *XbaI*. The digested PCR product and linearized vector were further purified and ligated using a T4 DNA ligase reaction mixture. The resulting construct was transformed into chemically competent TOP10 *Escherichia Coli* cells and the plasmid were isolated, purified, and confirmed via sequencing. The pGH19-mMct6 vector without the FLAG tag was generated using site-directed mutagenesis and used for all activity studies. The pGH19-hMCT6 vector was used and prepared similarly to that described previously [7].

### 2.4. Transfection of MCT6 in X. laevis Oocytes 

The transfection was performed similarly to in our previous publication [7]. Briefly, mMct6 and hMCT6 capped sense RNA (cRNA) was transcribed from *NotI*-linearized pGH19 vectors using the mMESSAGE mMACHINE T7 transcription kit. Approximately 13.8 nL of cRNA or water was injected into oocytes isolated from digested, resected ovaries the day before. The oocytes were then incubated in OR3 medium at 18 °C for 3 to 5 days. All cRNA was verified for purity, concentration, stability, and correct size. Due to previous unsuccessful attempts to detect our transporter of interest with commercially available antibodies, our lab generated transfected Mct6-FLAG-tagged oocytes using similar methods to verify the protein expression using an anti-FLAG antibody. Western Blotting was performed as done previously [7] on water-injected and Mct6-FLAG cRNA-injected oocytes from day 3 to day 5 post-injection (D.P.I). The concentration of anti-FLAG primary antibody was 1:1000.

### 2.5. Uptake Studies

The uptake studies were performed similarly to before [7]. Briefly, the uptake studies were performed using groups of 4 to 5 oocytes in 24-well multi-dishes and preincubated in uptake buffer for 30 min (15 mM 4-(2-hydroxyethyl)-1-piperazineethanesulfonic acid (HEPES), 82.5 mM NaCl, 2.5 mM KCl, 1 mM Na_2_HPO_4_, and 1 mM MgCl_2_, adjusted to pH 7.4 with Tris). For the time-dependent study, the oocytes were then transferred to 400 μL of uptake buffer containing 1 nM radiolabeled PGF2α ([^3^H]PGF2α), and the oocytes were incubated at pH 7.4 at room temperature (~20–23 °C). For the concentration-dependent study, PGF2α concentrations varied and the uptake time was chosen to be in the linear range of the time-dependent study. All uptake was stopped by the addition of ice-cold uptake buffer, and the oocytes were washed three times. Individual oocytes were placed in separate scintillation vials and dissolved in 250 µl of 10% sodium lauryl sulfate by slowly shaking for 1.5 h. Radioactivity was determined by liquid scintillation counting following the addition of a scintillation cocktail. All studies were performed with 4–5 oocytes for each data point, with experiments performed at least three separate times with at least 2 different ovaries.

For the *cis*-inhibition study, prototypical MCT6 substrates and other PGs were investigated for the inhibitory effects on murine and human MCT6-mediated PGF2α uptake (1 nM) at pH 7.4 and room temperature for 30 min. Two concentrations (10 µM and 0.1 µM) were investigated for each PG to investigate whether there was any concentration-dependent inhibition of MCT6-mediated uptake of PGF2α. Briefly, oocytes were either incubated in 1 nM PGF2α with or without other MCT6 substrates or PGs. MCT6-mediated PGF2α uptake was calculated as the difference between the MCT6 cRNA-injected oocytes and the water-injected oocytes.

### 2.6. Plasma and Urinary PGF2α and PGFM ELISAs in Mct6^+/+^ and Mc6^−/−^ Mice 

Blood and urine samples from Mct6^+/+^ and Mct6^−/−^ mice (15–25 weeks of age, N = 3–5) were collected following ad libitum feeding or fasting for 12–15 h overnight with free access to water. Plasma was isolated by centrifuging at 2000 × g for 10 min at 4 °C in heparinized tubes. Urine was spun at 10,000 × g for 5 min at 4 °C to pellet any insoluble material. All samples were stored at −80 °C until analysis. Samples were assayed for PGF2α and PGFM using commercially available ELISA kits according to the suggested manufacturer’s protocols (Cayman Chemical, Cat. # 516011, Cat. # 516671). Urinary PGF2α and PGFM were normalized to creatinine, which was measured using a commercially available kit according to the manufacturer’s protocol (Crystal Chem, Elk Grove Village, IL, Cat. # 80350).

### 2.7. Mct6 Gene Expression in Different Dieting States

Tissue preparation and gene expression analysis was performed as done previously [33]. Wild-type male mice were either fasted overnight (12–15 h) with free access to water or fed ad libitum. Briefly, kidney, liver, and colon tissues were harvested from wild-type mice (28–30 weeks of age, N = 4–5) and snap frozen in liquid nitrogen until RNA extraction. Colon was collected as 5 cm of large intestine following the cecum. A VWR Pellet Mixer was used to homogenize each tissue on ice, and total RNA was isolated and purified using a RNeasy Mini Kit (Qiagen) according to the manufacturer’s protocol. Concentration, purity, and stability were confirmed via a NanoDrop 1000 and FlashGel™ System. First-strand cDNA synthesis was performed using SuperScript^TM^ III RT (Thermo Fisher Scientific) according to the manufacturer’s protocol, and concentration and purity was further confirmed using the NanoDrop 1000. For the quantitative real-time polymerase chain reaction (qRT-PCR) analysis, Taqman^®^ gene expression assays were used for *Slc16a5* (Thermo Fisher Scientific, Assay ID: Mm01252138) and the housekeeping gene *Hprt* (Assay ID: Mm03024075). Cycle threshold (C_t_) were obtained from BioRad CFX Manager 3.0 software and imported into Excel for calculations.

### 2.8. Data Analysis

#### 2.8.1. Uptake Studies

The time-dependent PGF2α uptake rate by oocytes (μL/oocyte) was calculated as the ratio of radioactivity in each sample (dpm/oocyte) to the initial concentration in the uptake buffer (dpm/μL). All MCT6-mediated uptake was calculated as the difference between the MCT6 cRNA-injected oocytes and the water-injected oocytes. Data analysis was performed using GraphPad Prism 7 (GraphPad Software Inc., San Diego, CA, USA). The concentration-dependent PGF2α uptake rate (pmol/oocyte/30 min) was calculated as the ratio of radioactivity in each sample (dpm/oocyte) to the concentration in the uptake buffer (dpm/pmol). The kinetic parameters of concentration-dependent uptake of PGF2α was calculated by fitting with the equation below (Equation (1)) using weighted nonlinear regression analysis (ADAPT 5; Biomedical Simulations Research, University of South California, Los Angeles, CA, USA).

(1) $$J=\frac{J_{max}\cdot C}{K_{t}+C}$$

where *J* is the MCT6-mediated bumetanide uptake rate (pmol/oocyte/30 min), *C* is the concentration of bumetanide (µM), *J_max_* is the maximum uptake rate (pmol/oocyte/30 min), and *K_t_* is the substrate concentration at the half-maximal uptake rate (µM). For the *cis*-inhibition assay, data were expressed as the percentage of PGF2α uptake in comparison with the control (PGF2α alone).

#### 2.8.2. mRNA Expression

Data was analyzed in Excel using the 2^−ΔΔCt^ method. Data was normalized to mRNA expression in wildtype mice fed ad libitum and expressed as fold-change for each tissue.

#### 2.8.3. Statistical Analysis

All statistical analyses were performed using the one-way unpaired analysis of variance (ANOVA) followed by Dunnett’s test to test for multiple comparisons or an unpaired Student’s t-test. Differences were considered statistically significant when *p* < 0.05.

## 3. Results

### 3.1. Mct6 Protein Expression Is Stably Expressed in Transfected X. laevis Oocytes 

**Figure 1:**
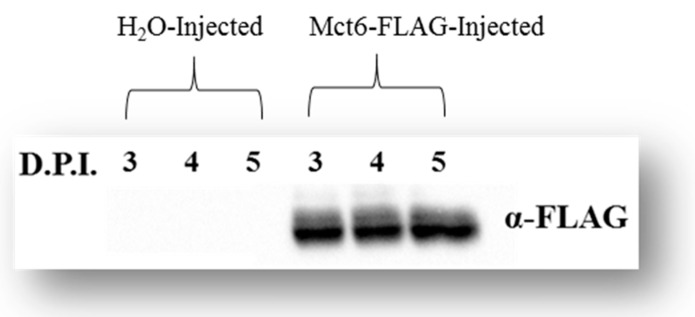
Western blot analysis demonstrating positive protein expression of Mct6-FLAG (~52 KDa) in transfected oocytes 3-5 D.P.I. using anti-FLAG (α-FLAG) primary antibody (D.P.I.: days post-injection).

To verify that Mct6 protein was expressed throughout the in vitro studies, Western blotting was performed on Mct6-FLAG cRNA-injected and water-injected oocytes from 3–5 days post-injection. Due to the lack of commercially available and reliable Mct6 primary antibodies, the FLAG-tagged protein was used as a substitute. Figure 1 depicts the presence of a ~52 KDa protein at similar intensity over the course of the study, which is consistent with the predicted molecular weight of a Mct6-FLAG conjugate. No bands were visible in water-injected oocytes.

### 3.2. Mct6 Transports PGF2α at Micromolar Affinity

**Figure 2:**
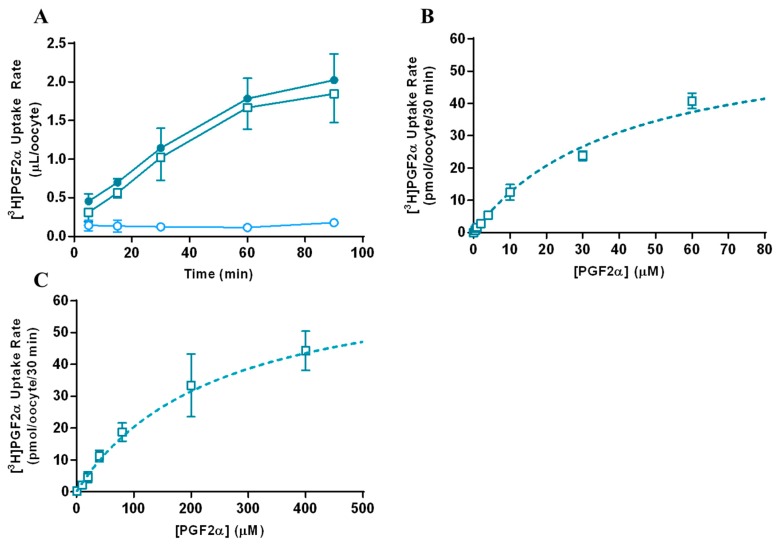
Time- (**A**) and concentration-dependent uptake of prostaglandin F_2α_ (PGF2α) in murine monocarboxylate transporter 6 (mMct6) (**B**) and human monocarboxylate transporter 6 (hMCT6) (**C**)-transfected *Xenopus laevis* oocytes. (N = 4–5 oocytes/data point). Experiment was performed at least three separate times with at least 2 different ovaries. Data are presented as mean ± standard deviation (SD). Closed circles: MCT6 cRNA-injected, open circles: water-injected, open squares: MCT6-mediated uptake (MCT6 cRNA-injected minus water-injected). Dashed line represents model fitting using Equation (1) to MCT6-mediated uptake data.

The results from the time- and concentration-dependent uptake of PGF2α are depicted in Figure 2. Uptake of 1 nM PGF2α at pH 7.4 and room temperature (r.t.) was in the linear range of uptake (Figure 2A) prior to steady state at approximately 60 min. Therefore, for the purpose of the concentration-dependent uptake studies, 30 min was used as the uptake time. By fitting with Equation (1), the transporter kinetic parameters (K_t_, J_max_) for mMct6-mediated (Figure 2B) and hMCT6-mediated (Figure 2C) transport of PGF2α were calculated. For mMct6 at pH 7.4, the affinity (K_t_) was 40.1 ± 4.33 μM and the maximal capacity (J_max_) was 62.3 ± 7.78 pmol/oocyte/30 min. For hMCT6 at pH 7.4, the K_t_ was 246 ± 65.2 μM and the J_max_ was 70.3 ± 15.1 pmol/oocyte/30 min.

### 3.3. Mouse and Human MCT6-Mediated PGF2α Transport Are Inhibited by Other Eicosanoids and MCT6 Substrates

To investigate the effects of other eicosanoids on the activity of murine and human MCT6-mediated PGF2α transport, *cis*-inhibition assays were performed using various concentrations (0.1 and 10 µM) of PGs (prostaglandin E2 (PGE2), prostaglandin D2 (PGD2), and 6-keto prostaglandin F_1α_). Bumetanide and probenecid were also investigated as inhibitors of Mct6-mediated transport.

**Figure 3:**
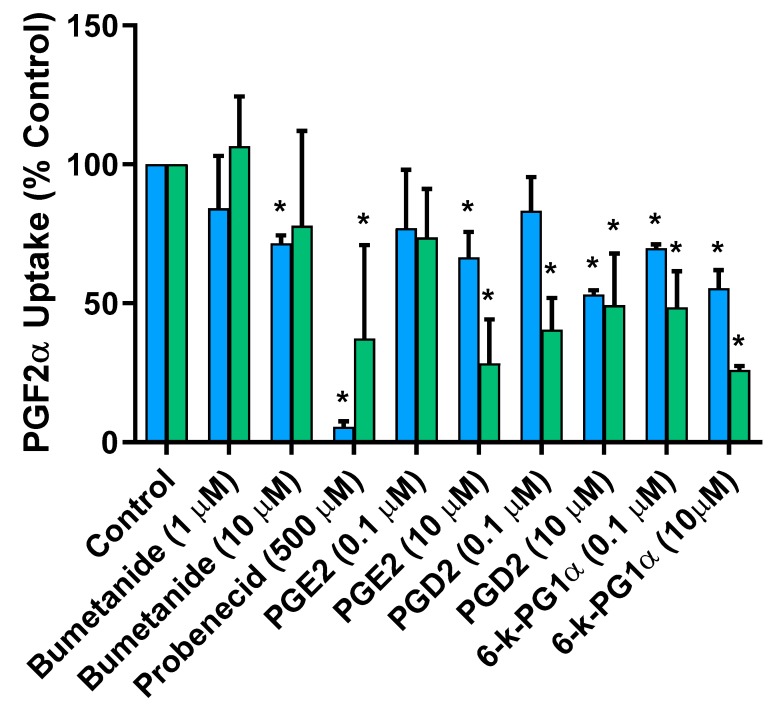
*Cis*-inhibition assay demonstrating the inhibition of prostaglandin F_2α_ (PGF2α) uptake by prototypical monocarboxylate transporter 6 (MCT6) substrates (bumetanide and probenecid) as well as other members of the eicosanoid family (PGE2: prostaglandin E2, PGD2: prostaglandin D2, 6-k-PG1α: 6-keto prostaglandin F1α) in murine Mct6 (blue) and human MCT6 (green)-transfected oocytes. Data are presented as mean percent control uptake in the absence of inhibitor ± SD. Experiments performed using 1 nM PGF2α, pH 7.4, r.t., for 30 min (* *p* < 0.05 compared to control).

As shown in Figure 3, both 10 µM bumetanide and 500 µM probenecid significantly inhibited murine Mct6-mediated uptake (~28% and 94% inhibition, respectively), as expected. Most inhibitors were evaluated at high concentrations that were >100-fold higher than their reported unbound plasma concentrations. Bumetanide and probenecid also inhibited human MCT6-mediated PGF2α uptake, and all the PGs inhibited PGF2α uptake to some extent (~26–59% inhibition for 0.1 µM inhibitor concentration, ~51–74% inhibition for 10 µM inhibitor concentration). The PGs screened in this assay were more potent inhibitors of human MCT6-mediated PGF2α uptake than murine Mct6-mediated uptake.

### 3.4. Plasma and Urinary PGF2α and PGFM Concentrations in Mct6^+/+^ and Mc6^−/−^ Mice Are Significantly Altered, and Mct6 Exhibits a Diet-dependent Gene Expression Profile 

**Figure 4:**
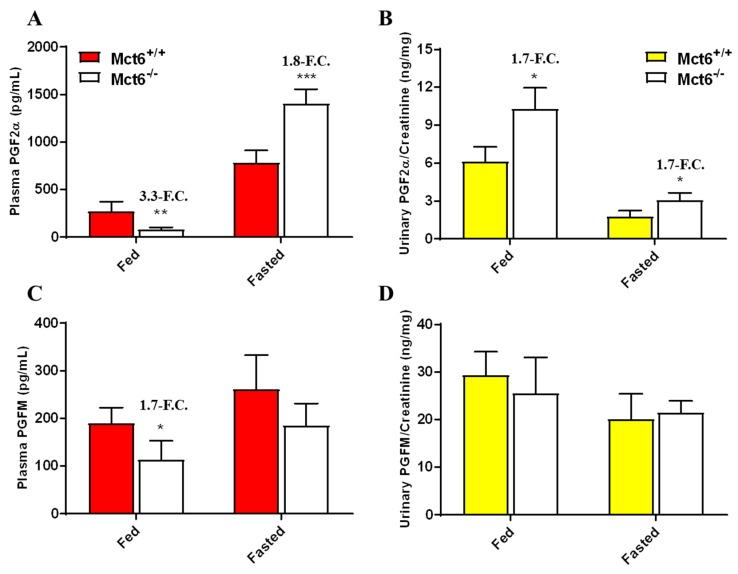
Plasma (**A**/**C**) and urinary (**B**/**D**) PGF2α and PGFM concentrations in Mct6^+/+^ and Mc6^-/-^ mice. Mice were 15–25 weeks of age (N = 3–5 per group). Urine values were normalized to creatinine concentration. Data are plotted as mean ± SD. (F.C.: fold change). Statistical comparisons are made between Mct6^+/+^ versus Mct6^−/−^ mice (* p < 0.05; ** p < 0.01; *** p < 0.001).

Considering PGF2α was confirmed as a substrate for human and murine Mct6, our lab investigated whether or not endogenous levels of PGF2α, as well as 13,14-dihydro-15-keto PGF_2α_ (PGFM), a primary metabolite of PGF2α, were altered between the Mct6^+/+^ and Mct6^−/−^ mice. As shown in Figure 4, there were significant changes between the Mct6^+/+^ and Mct6^−/−^ mice dependent on dieting state. Mct6^−/−^ mice fed ad libitum exhibited significantly decreased PGF2α (3.3-fold) and decreased PGFM (1.7-fold) plasma concentrations in comparison to Mct6^+/+^ mice. Inversely, fasted Mct6^−/−^ mice exhibited significantly increased PGF2α (1.8-fold) plasma concentrations in comparison to Mct6^+/+^ mice. Independent of dieting state, PGF2α urinary concentrations normalized to creatinine were elevated (1.7-fold). No change in urinary PGFM was detected between the two groups in either dieting state.

**Figure 5:**
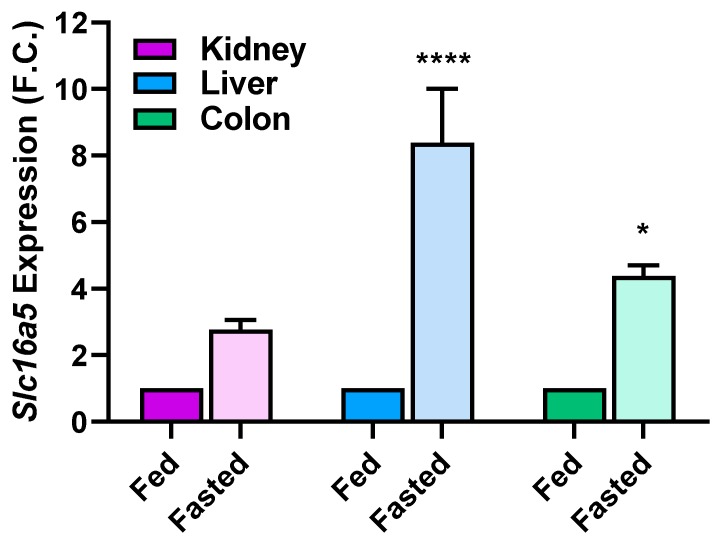
mRNA expression of *Slc16a5* (Mct6) in fed and fasted diets in Mct6^+/+^ mice. All gene expression data were normalized to a housekeeping gene (*Hprt*). Data were plotted as mean fold change ± standard error of the mean (SEM) (N = 4–5 mice, 28–30 weeks of age). Gene expression was normalized to fed dieting state for each tissue (**** *p* < 0.0001, * *p* < 0.05, compared to the fed dieting state for each tissue).

With regards to changes in mRNA expression, Figure 5 depicts the relative changes in Mct6 gene expression in fasted versus fed dieting states. *Slc16a5* expression increased in the kidney, liver, and colon following an overnight fast. Most prominently, Mct6 gene expression increased 8.4-fold in liver in a fasted state compared to a fed state and this change was significantly higher than that of the kidney and colon. In addition, colonic gene expression of Mct6 was significantly increased in the fasted state compared to the fed state (4.4-fold).

## 4. Discussion

The purpose of this study was to investigate the role of MCT6 in PGF2α transport and to assess whether this transporter alters PGF2α concentrations in vivo using a Mct6 knockout mouse model. A previous study performed by Murakami et al. in 2005 suggested that PGF2α is transported by human MCT6; however this study was only performed at one concentration and the kinetics were not characterized [5]. In addition, there is substantial evidence in the literature suggesting that murine Mct6 is markedly upregulated in different dieting scenarios [10,11], as well as studies by our lab showing that Mct6-mediated transport is inhibited by a variety of diet-based aglycone flavonoids [7]. Therefore, our current goal was to determine whether or not Mct6 could play a role in the homeostasis of PGF2α, as well as potentially other PGs, and if these regulatory processes were diet-dependent.

### 4.1. In Vitro Kinetics of MCT6-Mediated PGF2α Transport

Previously, our lab investigated effects of human MCT6-mediated transport of bumetanide, a loop diuretic substrate of MCT6, and the effects of various dietary flavonoid interactions [7]. In a similar manner, our lab aimed to characterize the MCT6-specific transporter kinetic parameters (K_t_ and J_max_) of PGF2α using human and murine MCT6-transfected *X. laevis* oocytes. The expression of FLAG-tagged murine Mct6 was stable in *X. laevis* oocytes 3–5 days post-injection. Similar studies were performed previously using a human MCT6-Egfp-tagged construct, which also showed similar stability over the course of our uptake studies [7]. The calculated affinity (K_t_) for murine Mct6 was 40.1 µM, while the affinity for human MCT6 was much less at 281 µM at pH 7.4. Due to previous findings suggesting that nateglinide and bumetanide (both previously characterized substrates for MCT6) had different binding sites due to their noncompetitive inhibition profile [5], we believe that the species differences between the mouse and human affinities for PGF2α may be accounted for, in part, by the multiple binding sites of MCT6. It is also important to note that endogenous concentrations of PGF2α in mice and humans range from 100 to 700 pg/mL, which translates to approximately 0.28–2 nM [35,36]. Other PG transporters, such as organic anion transporters OATP, OAT, and MRP families, have all been shown to transport PGF2α with a much higher affinity (in the low micromolar-nanomolar range) than MCT6, although there are some conflicting reports as to the specific affinity for each transporter and species-specific isoforms [16,21,25,37]. However, the concentrations of PGs or their structurally similar polyunsaturated fatty acid precursors are expected to be much higher in the intestinal lumen, where MCT6 protein is highly expressed, so transport of these molecules by MCT6 may potentially be of importance in the gastrointestinal tract. While abundance of different fatty acids tend to vary widely depending on diet, some reports have shown that these fatty acids can range from the low to mid millimolar concentrations [38], which suggests that MCT6 would not be saturated under these conditions. In particular, for omega-6 polyunsaturated fatty acids, which are precursors for PG 2-series compounds, these concentrations can also vary dependent on their dietary sources [39].

Upon investigating whether other members of the eicosanoid family, as well as other substrates for human MCT6 interacted with murine Mct6, a *cis*-inhibition assay revealed that many other structurally similar PGs significantly inhibited MCT6-mediated PGF2α uptake. Most inhibitors were evaluated at much higher concentrations that were >100-fold higher than their reported unbound plasma concentrations. Because PGF2α is a recognized Mct6 substrate, and all other PGs investigated in this analysis are 20-carbon-containing long chain fatty acids similar to PGF2α, it is expected that these compounds are potentially substrates, although additional uptake studies with these compounds will be needed to confirm this.

### 4.2. Diet-Dependent Evaluation of PGF2α and PGFM Concentrations in Mct6^+/+^ and Mct6^−/−^ Mice

Members of the PG 2-series are largely synthesized ubiquitously in every tissue via the arachidonic acid metabolism-associated cyclooxygenase (COX)-mediated pathways [40]. PGF2α, in particular, has been demonstrated to be secreted and metabolized via highly vascularized tissues such as the lungs [41,42], reproductive tissues [43], as well as the kidneys [44,45]. To confirm our hypothesis that Mct6 plays an endogenous role in regulating the availability of PGF2α, plasma and urinary concentrations of PGF2α, as well as its primary metabolite (PGFM) were measured in Mct6^+/+^ and Mct6^-/-^ mice. In addition, due to evidence suggesting dietary regulation of Mct6, these studies were performed in two different dieting states (ad libitum feeding and overnight fasting) in order to evaluate the effects of diet on Mct6′s regulatory role of PGF2α. Our findings suggested that not only did Mct6 regulate systemic and urinary concentrations of PGF2α, but this was also dependent on the dieting state. A diet-dependent role was characterized for MCT1 in mice, which showed that haplo-insufficient MCT1 mice displayed resistance to diet-induced obesity [46]. In agreement with what was previously reported in the literature, overnight fasting largely induced Mct6 gene expression in mouse liver [11]. Also, fasting significantly increased Mct6 gene expression in the colon as well; however, the largest changes in Mct6 gene expression were seen in the liver (liver > colon > kidney). Note that for the purpose of the gene expression profile diet comparison study, *Hprt* was used as the reference gene considering it has been demonstrated to be a suitable reference gene when comparing across different diets in mouse and rat tissues [47,48]. No significant difference in *Hprt* expression was seen when comparing the two diets. It is important to note that other MCT isoforms have also been reported to be upregulated in fasted versus a fed state in metabolic tissues such as the liver and kidney; however, these changes tend to be isoform- and tissue-specific [47]. Considering we know that fasting induces Mct6 gene expression in the liver, a major site for β-oxidation and PG elimination, and that in a regularly fed state Mct6 expression is apparently highest in the intestine in comparison to other tissues, the discrepancy between PGF2α plasma concentrations in different dieting states may be due to tissue-specific roles regulating systemic prostaglandin availability. Additionally, it is important to note that PGF2α plasma concentrations were significantly increased in the fasted versus fed diet to a greater magnitude in Mct6^−/−^ mice compared to wild-type mice. Therefore, the impact of feeding on PGF2α homeostasis was greater in the absence of Mct6 activity. Further studies are necessary prior to mechanistically understanding the reasons for this phenomenon. In the fed state, Mct6 may play a role in the biosynthesis of PGF2α, possibly due to intestinal absorption of precursors such as omega-6 polyunsaturated fatty acids, while its role in fasted states may be different. Decreases in PGFM in the plasma may also reflect the decreased availability of parent PGF2α in the fed state. The elevated urinary PGF2α concentrations in both diets, however, reveal a diet-independent Mct6-mediated role in regulating PGF2α in the urine. This increase in urinary PGF2α may suggest a role of Mct6 in active renal reabsorption or secretion; however, this needs to be further investigated. In addition, the lack of changes seen in urinary PGFM concentrations could be explained due to the fact that renal clearance is a minor route of elimination for PGFM [49].

Diet-specific regulation of Mct6 via PPAR-dependent pathways may also partly explain the large changes in expression seen following a fasted diet [50]. Previous gene and protein expression profiling performed by our lab has demonstrated that the principal network generated using bioinformatics analysis revealed changes in *Pparα* expression, a key transcription factor in modulating hepatic lipid homeostasis [33]. While there were significant changes between the wild-type and Mct6^−/−^ mice associated with lipid metabolism, there were no significant differences reported in major enzymes involved in PG elimination or synthesis, such as dehydrogenase and synthase/reductase enzymes. Additionally, Xu et al. suggested the involvement of butyrate-mediated *Pparγ* activation on Mct6 regulation in diabetic rats [12]. This suggests that Mct6 is regulated, in part, via PPAR-dependent mechanisms, which play a role in regulation of lipid metabolism, and effectively, PG homeostasis. However, further investigation with potent PPAR inhibitors/inducers is necessary prior to confirming their important in Mct6 regulation.

In conclusion, our findings confirmed that PGF2α is an MCT6 substrate and that murine Mct6 significantly contributes to systemic and urinary concentrations of PGF2α, in a diet-dependent manner. These results are the first to suggest the role of MCT6 in PGF2α homeostasis, as well as potentially other PGs, in distribution and metabolism. This research also utilized the first experimental clustered regularly interspaced short palindromic repeats/CRISPR associated protein 9 (CRISPR/Cas9) Mct6^−/−^ mouse model, which may help reveal Mct6′s functional role in lipid metabolism. The clinical impact and relevance of these findings are expected to be novel and useful, considering PGF2α is an important mediator of many physiological phenomena such as inflammation, adiposity, and others. Lastly, these discoveries have endorsed the possible deorphanization of MCT6, annotating its physiological role in PG homeostasis and lipid metabolism, potentially opening up new windows of opportunity for its utility as a clinical target in disease.
